# Recurrence in a patient with a 10-year history of sinonasal mucosal melanoma manifesting as facial swelling

**DOI:** 10.4317/jced.54466

**Published:** 2017-12-01

**Authors:** Eleni-Marina Kalogirou, Demos Kalyvas, Konstantinos I. Tosios, Kostas Tsiklakis, Alexandra Sklavounou

**Affiliations:** 1 DDS, MSc, PhD Candidate, Department of Oral Medicine and Pathology, Faculty of Dentistry, National and Kapodistrian University of Athens, Athens, Greece; 2 DDS, PhD, Associate Professor, Department of Oral and Maxillofacial Surgery, Faculty of Dentistry, National and Kapodistrian University of Athens, Athens, Greece; 3 DDS, PhD, Assistant Professor, Department of Oral Medicine and Pathology, Faculty of Dentistry, National and Kapodistrian University of Athens, Athens, Greece; 4 DDS, MSc, PhD, Professor, Department of Oral Diagnosis and Radiology, Faculty of Dentistry, National and Kapodistrian University of Athens, Athens, Greece; 5 DDS, MSc, PhD, Professor, Department of Oral Medicine and Pathology, Faculty of Dentistry, National and Kapodistrian University of Athens, Athens, Greece

## Abstract

Sinonasal mucosal melanoma is a rare tumor that develops slowly and may manifest with non specific signs and symptoms, causing significant delay in diagnosis. Local recurrence is common and usually occurs within the first two years after the initial treatment. Prognosis of recurrent lesions is poor and 5-year survival ranges between 10-47%. We report the clinical, radiographic, histopathological and immunohistochemical findings of a recurrent sinonasal mucosal melanoma which was diagnosed 10 years after the initial treatment, in a patient who presented with unilateral facial swelling and one-sided difficulty in breathing of two years duration. We discuss the causes of late diagnosis and review the negative predictive factors for recurrence and survival. As early diagnosis is of paramount importance for prognosis, we emphasize the signs and symptoms of patients with a history of sinonasal mucosal melanoma which should raise the suspicion for recurrence, in spite of a long time interval since diagnosis.

** Key words:** Mucosal melanoma, nasal cavity, sinus, recurrence.

## Introduction

Sinonasal mucosal melanoma (SNMM) accounts for 0.3-2% of all primary malignant melanomas (1), approximately 2-9% of head and neck mucosal melanomas (2) and 4-8% of all sinonasal malignancies (3). It affects patients in 5th to 8th decade of life (4-6), generally older than those with cutaneous melanoma (1), probably because of an increase in the number of melanocytes with advancing age (7). There is no definite gender predilection (1,4,8).

Most primary SNMMs (65.5-80.9%) arise within the nasal cavity (5,6), in particular in the nasal septum, the inferior and middle turbinate, the lateral nasal wall and the nasal floor (3,4,6,8,9). The paranasal sinuses are less frequently involved, usually the maxillary sinuses (15.1-16.9%), followed by the ethmoid sinus, the frontal sinus and sphenoid sinus (5,6). The predominant manifestations of SNMMs are unilateral epistaxis and nasal obstruction (3,6,8,9). Symptoms vary according to tumor size and location (8) and advanced cases may present with enlargement causing nasal or facial deformity, facial pressure, pain, and less often frontal headache, hypoesthesia, unilateral hearing loss, rhinorrhea, hyposmia, congestion, breathing discomfort, diplopia, proptosis and epiphora (1,3,7-9). The indolent and non specific clinical appearance of SNMM may lead to a significant delay in diagnosis (1,8,9), which affects negatively the prognosis of the disease (9).

We present a recurrent mucosal melanoma diagnosed 10 years after the initial diagnosis, with unilateral facial swelling and difficulty in breathing of two years duration, and discuss the causes of the delay in diagnosis. Prognostic factors affecting the recurrence and survival rate are also reviewed.

## Case Report

A 65-year-old female was referred by her dentist for evaluation and management of right facial swelling, with a provisional diagnosis of a “maxillary cyst”. According to the patient, the swelling was initially noticed approximately 2 years before presentation, grew slowly and was associated with unilateral difficulty in breathing. Her medical history was significant for a primary mucosal melanoma of the right nasal turbinate treated 10 years ago with surgical excision without adjuvant radiotherapy or chemotherapy. The patient did not attend a regular follow-up program. She did not take medications; she did not smoke and her recent complete blood count did not reveal any abnormality.

**Figure 1:**
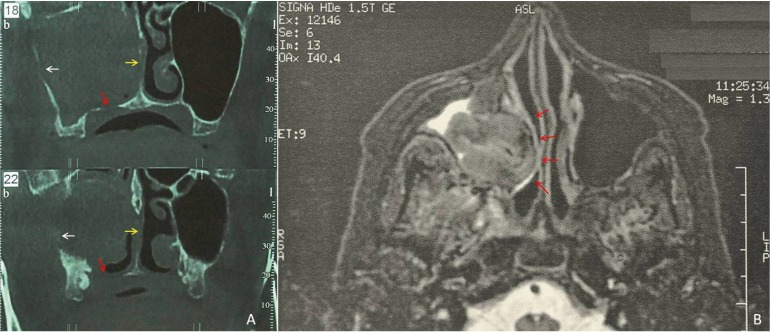
(a) Coronal Computer Tomography showed a solid, voluminous, hypointense lesion occupying the middle and inferior turbinate and the whole right sinus cavity, with thickening of the sinus mucosa and perforation of the sinus floor (red arrow) and the lateral nasal wall (yellow arrow). (b) A prominent expansion of the lateral nasal wall was observed on the magnetic resonance imaging (red arrows).

**Figure 2:**
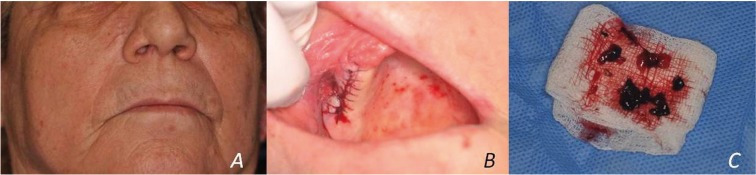
(a) Extraoral examination revealed a mild, diffuse swelling in the middle right side of face. (b) Incisional biopsy was performed under local anesthesia using a transoral approach and (c) revealed dark black colored surgical specimens.

Intraoral examination was within normal limits. A coronal Computer Tomography plane showed a solid, voluminous, hypointense lesion occupying the middle and inferior turbinate and the whole right sinus cavity, with thickening of the sinus mucosa and perforation of the sinus floor (red arrow) and the lateral nasal wall (yellow arrow) (Fig. 1a). A prominent expansion of the lateral nasal wall was observed on the magnetic resonance imaging (Fig. 1b, red arrows) and was considered consistent with the reported breathing discomfort. Extraoral clinical examination revealed a mild, diffuse swelling in the middle right side of face (Fig. 2a). With the provisional diagnosis of a recurrent melanoma, an incisional biopsy was performed using a transoral approach (Fig. 2b) under local anesthesia. The surgical specimens were black in color (Fig. 2c) and measured 2x0.7x0.3cm in aggregate.

**Figure 3:**
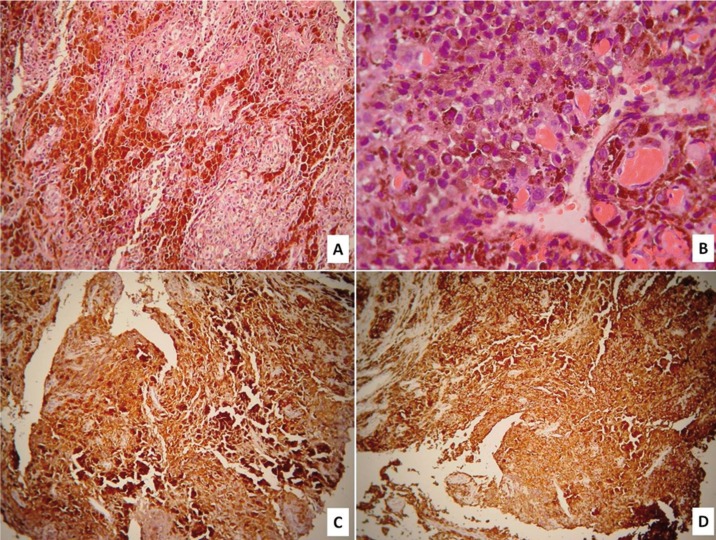
Histopathologic and immunohistochemical examination: (a) Spindle and epithelioid tumor cells, arranged in sheets or solid nests, (b) with large, pleomorphic, hyperchromatic/vesicular nuclei and abundant, eosinophilic/hypochromatic cytoplasm. Strong immunopositivity for (c) Melan-A/Mart-1 and (d) HMB-45 [(a,b)hematoxylin-eosin stain; (c,d) streptavidin-biotin-peroxidase; original magnifications (a,c,d)x100, (b)x400].

Histopathologic examination of 5μm thick formalin-fixed and paraffin-embedded tissue sections stained with hematoxylin and eosin showed a tumor composed of spindle and epithelioid cells arranged in sheets or solid nests (Fig. 3a). Neoplastic cells ex-hibited large, pleomorphic, hyperchromatic or vesicular nuclei with multiple, prominent nucleoli and abundant eosinophilic or hypochromatic cytoplasm with intracytoplasmic brown pigment aggregates that were consistent with melanin (Fig. 3b). Approximately 10 mitotic figures were noted per high power field. Lymphocyte, plasma cells and polymorphonuclear neutrophil infiltrates, areas of hemorrhage and melanin granules were seen within the vascular stroma. Extensive tumor necrosis and foci of amorphous eosinophilic material were also observed. Tissue sections were treated with monoclonal antibodies against Me-lan-A/Mart-1 (clone A103, dilution 1:25, Dako, Glostrup, Denmark) and HMB-45 (clone MO643, dilution 1:50, Dako), using a standard streptavidin-biotin-peroxidase system and the Dako EnvisionTM system (Dako). Α strong, diffuse positivity for Melan-A/Mart-1 (Fig. 3c) and HMB-45 (Fig. 3d) was detected. Based on the histopathological and immunohistochemical findings, a diagnosis of a recurrent SNMM was rendered. The patient underwent a full course of chemotherapy, but she died of her disease 18 months later. At the time of biopsy, the patient gave informed consent for the future use of her data for study.

## Discussion

In the case presented herein the indolent growth of a recurrent SNMM caused significant delay in diagnosis. Primary SNMM develops slowly (9); its clinical features are non-specific and usually precede diagnosis up to 24 months (3,6). Growth of the recurrent lesion into the sinus cavity may have contributed to the non-specific manifestations, such as facial swelling, which usually indicates a late stage of the disease (10). In a clinicopathological study of 44 SNMMs (3), outspread of the recurrent lesion to the paranasal sinuses was seen in 29.55%, and within the nasal cavity in 34.09%. Mucosal melanomas of the sinus usually grow silently, become evident at late stages (4,5) and bear a significantly poorer prognosis compared to the nasal mucosal melanomas (3,4), due to delayed diagnosis and anatomical variations that complicate the surgical access (11). The unilateral difficulty in breathing probably results from nasal obstruction that is among the most frequent symptoms of SNMM (3,6,8,9) and is considered an unfavorable prognostic factor when it is not associated with epistaxis (1).

In our case, diagnosis was also delayed by the underestimation of the patient’s history, possibly because of the long time interval between the initial diagnosis of the SNMM and its recurrence. Local recurrence is expected in 31%-85% of patients (4,9) and the average relapse time usually ranges from 9 to 25 months (3,4). However, recurrence may occur even 10 years after the initial therapy, directly followed by multiple organ metastases and, as in our patient, a fatal outcome (1). The long-term interval from initial diagnosis until recurrence is more probable in SNMMs involving the paranasal sinuses (12).

Several clinical, i.e. advanced age at initial diagnosis, isolated obstructive phenomena and atypical manifestations, including headache, facial pain and maxillary nerve anesthesia, anatomic, i.e. origin within the paranasal sinuses, and histopathological parameters, such as the presence of ≥10 mitoses per high power field, increased level of melanotic pigmentation, pseudopapillary or sarcomatoid architecture, perineural or vasolymphatic invasion, undifferentiated cell morphology, multiple, satellite formation and a positive margin status at initial surgery, have been associated with an increased risk for locoregional recurrence or/and a poor survival in SNMM (1,3,4,6,12). In the current case, recurrence within the sinus cavity (3,4), difficulty in breathing without epistaxis (1,3), and the advanced age of the patient (1,6), were negative prognostic factors.

Frequent and regular follow-up sessions for patients with a SNMM history, i.e. every 2-4 months, is recommended for at least the first decade post treatment (1), as early diagnosis and timely management of recurrences contribute to a longer survival (4). The five-year overall survival is poor and ranges between 10-47% (4,5,10), while 10-year survival decreases approximately at 20-25% (1,10). Close monitoring, early diagnosis of recurrence and prompt management is of outmost importance for prolonged survival (1,4,10). Surgical excision remains the treatment of choice, while radiotherapy, chemotherapy, biotherapy and molecular targeted may have a positive effect on recurrence rate (12), without however increasing the overall survival (3,13). Chemotherapy is suggested in advanced cases and in recurrent SNMMs (9) that are more infiltrative or multilocular compared to the primary tumor, and may pose difficulty for complete surgical resection (14).

In conclusion, the non-specific clinical manifestations, such as facial edema and breathing discomfort, in a patient with a history of SNMM should raise the suspicion of recurrent tumor, in spite of a long time interval from initial diagnosis.
